# In Memoriam—Edgar Abbott Ballard, M. D.

**Published:** 1892-01

**Authors:** H. Hitchcock


### IN MEMORIAM—EDGAR ABBOTT BALLARD, M. D.

DIED NOV. 6TH, 1891, AT HIS RESIDENCE, NO. 97 37TH STREET, CHICAGO, ILL.

Edgar Abbott Ballard was born at Williamsburg (now Brooklyn, E. D.) New York, March 8th, 1838. His early years were passed in Massachusetts, where he received the limited instruction afforded by the district schools of that time. With this limited education he went to New York City and there for a time engaged in mercantile pursuits. Later he adopted the theatrical profession, and acquired quite a reputation as a comedian, playing in various parts of the country. During an engagement in Memphis, Tenn., in 1859 he was prostrated by inflammation of the bowels, following an attack of dysentery which brought him to death's door, and he was sent home December 2d, 1859, in a most precarious condition. He became the charge of the late Dr. George E. Shipman, of Chicago, receiving from him his first dose of the homœopathic remedy, and, as he tersely stated it, "was snatched from the grave of disease and allœopathy, *principally the latter*."

The rapid improvement following Dr. Shipman's treatment, and the rational doctrines and principles which he inculcated made so deep an impression on the patient that he immediately devoted his entire energies and ability to master the science and art of healing. With Dr. Shipman as his preceptor he entered the Hahnemann Medical College of Chicago, from which he was graduated in 1863. With the exception of about eighteen months he has since practiced his profession in Chicago.

In 1870, after having diligently studied the question of the high potencies, feeling dissatisfied with the results obtained by the use of the lower ones, he adopted them exclusively, and has since then conscientiously used them with a comprehension of their powers and a knowledge of their effects seldom attained and rarely excelled. *Apropos* of a recent discussion he lately wrote "I am a living lie to the statement that Homœ-

opathy is incapable of mastering malaria, for I have successfully treated congestive chills from the swamps of Louisiana and have yet to give my first dose of quinine."

Dr. Ballard was one of the few consistent followers of Hahnemann who ever with might and main fought against the encroachment on pure scientific treatment of the empirical methods of the allœopathic and eclectic schools of medicine, and he spoke with no uncertain voice, nor did he seek to palliate one iota the force of his words when their keen incisiveness seemed harsh against those who assumed to practice according to homœopathic principles yet constantly resorted to un-homœopathic measures, and sought conciliation with the empirical physico-chemical schools. Thus in 1880, when the American Institute of Homœopathy had so far departed from its original purposes that Homœopathy was all but eliminated from its proceedings, he was one of the few who withdrew from it, and organized the International Hahnemannian Association. This Association has now become the hope and salvation of Homœopathy, and it recognized in Dr. Ballard one of its strongest supports. He was its Secretary for 1887–88, when failing health compelled him to resign further duties, and in 1889 the members of the Association at its meeting in Toronto, Canada, presented him with an elegant testimonial in appreciation of his personal worth and his long and ardent services in behalf of pure Homœopathy in Chicago, and in 1890, at the meeting of the Association at Watch Hill, Dr. Ballard was enrolled with Dr. P. P. Wells, of Brooklyn, at the head of the list of Honorable Seniors of that body.

By the death of Dr. Ballard the Association loses one of its most ardent friends and earnest supporters, and the profession one of its most able members, whose place will be hard to fill.

In 1864 he married Miss Elizabeth B. Huntley, who survives him, with a daughter and son. His brothers, Henry E. and W. A. Ballard, and sister, Mrs. C. O. Wilder, also his aged mother, are still living. For many years he was a great sufferer, and finally, in 1880, he was obliged to submit to an operation for the removal of his left foot, and since then never fully recovered his strength.

In private life Dr. Ballard was one of the most companionable of men. His quick wit and ready repartee were sure to enliven the spirits and quicken the thoughts of those about him. He was broad and liberal-minded, a humanitarian in the widest sense of the word, kind and sympathetic to the suffering, ready and willing at all times to give aid and counsel, a true friend indeed. Not alone will he be missed at the family fireside, but hundreds of those whose sufferings he had assuaged, those who have met him in social intercourse, his professional brethren, who loved him not less for himself than for his superior abilities, will sincerely mourn their loss, and keenly feel the absence of his cheery voice and kindly eye. HITCHCOCK.

